# Supplementary material for: WordCommentsAnalyzer: A windows software tool for qualitative research
Source: F1000Res. 2018 Sep 4;7:536. Originally published 2018 May 3. [Version 2] doi: 10.12688/f1000research.14819.2 (PMC6137411; doi:10.12688/f1000research.14819.2)
Supplement: Supplementary file 3 [file f1000research-7-17614-s0002.tgz › b2a79496-4ada-47c4-9682-a99ce867836d.zip!/Eight Iranian Health Journals/Journal of Knowledge and Health_10.docx]

© Journal of Knowledge and Health. This work is licensed under CC BY 4.0 (https://creativecommons.org/licenses/by/4.0/).

Volume & Issue: دوره 10، شماره 4: 1394

Title: بررسی اثر خاصیت ضدمیکروبی عصاره‌های الکلی گل محمدی و آلوئه‌ورا بر باکتری اشرشیاکلی

http://knh.shmu.ac.ir/index.php/site/article/view/769

مقدمه: عصاره‌ها و اسانس‌های گیاهی در زمینه پزشکی و صنایع غذایی کاربرد فراوانی دارند و دارای خاصیت ضدمیکروبی در برابر طیف وسیعی از میکروارگانیزم‌ها می‌باشند. در این تحقیق اثر ضدمیکروبی عصاره‌های اتانولی گل محمدی و آلوئه‌ورا نسبت‌به باکتری اشرشیاکلی مورد بررسی قرار گرفت. مواد و روش‌ها: عصاره‌گیری به‌روش خیساندن انجام گرفت، پس از تهیه عصاره‌ها، برای تعیین اثر ضدمیکروبی عصاره‌های اتانولی گل محمدی و آلوئه‌ورا نسبت‌به باکتری E.coli از روش انتشار در آگار diffusion) (Agar استفاده شد و حداقل غلظت مهارکنندگی (MIC) با استفاده از روش رقت لوله تعیین گردید. نتایج: نتایج نشان داد که عصاره اتانولی گل محمدی نسبت‌به عصاره اتانولی آلوئه‌ورا اثر ضدمیکروبی بیشتری در برابر باکتری اشرشیاکلی دارد. قطر هاله و حداقل غلظت مهارکنندگی برای عصاره اتانولی گل محمدی به‌ترتیب برابر 14mm و g/mlµ25MIC=  و قطر هاله و حداقل غلظت مهارکنندگی برای عصاره اتانولی آلوئه‌ورا به‌ترتیب برابر 7mm و g/mlµ50MIC=  بود. نتیجه‌گیری: عصاره گل محمدی نسبت‌به عصاره آلوئه‌ورا دارای اثر ضد‌میکروبی بیشتری بوده است همچنین ترکیب عصاره اتانولی گل محمدی و آلوئه‌ورا باعث کاهش خاصیت ضدمیکروبی این عصاره‌ها گردید.

Title: مهار موقت بیان ژن VEGFR2 با استفاده از siRNA اختصاصی در محیط کشت

http://knh.shmu.ac.ir/index.php/site/article/view/786

مقدمه: استفاده از siRNA برای خاموش‌سازی بیان ژن‌ها امروزه در حال گسترش است. از جمله اهداف این تکنولوژی؛ جلوگیری از بیان عامل رشد اندوتلیال عروقی است. مطالعه حاضر به‌منظور جلوگیری بیان گیرنده تیپ II این عامل (VEGFR-2: Vascular Endothelial Growth Factor Receptor II) با استفاده از siRNAی اختصاصی در محیط کشت صورت گرفته است. مواد و روش‌ها: ابتدا با استفاده از توالی ژن هدف، توالی‌های siRNAی اختصاصی علیه آن طراحی؛ Blast و ساخته شد. از طرف دیگر cDNA سلول HUVEC سنتز و با کمک پرایمر اختصاصی و PCR به میزان موردنیاز تکثیر شد و سپس در وکتور بیانی PEFGP-N1 درج و تأيید شد. پلاسمید ناقل حاصله با استفاده از لیپوفکتامین به سلول Hela که فاقد بیان ژن هدف بود انتقال داده شد. میزان بیان  GFPدر وکتور اولیه و وکتور تراریخت شده در حضور و عدم حضور siRNA اختصاصی مورد ارزیابی قرار گرفت و میزان مهار ژن از طریق ارزیابی کاهش فلورسانس سبز رنگ ناشی از GFP و RT-PCR مورد بررسی قرار گرفت. نتایج: نتایج حاصل از دو siRNA به‌کار رفته بیانگر کاهش بیان ژن به‌ترتیب 56% و 62% در مقایسه با گروه کنترل بود (05/0P<). نتیجه‌گیری: siRNAی اختصاصی طراحی شده علیه VEGFR-2 با استفاده از لیپوفکتامین به‌طور مناسب به‌داخل سلول منتقل و از بیان گیرنده به‌طور معنی‌دار جلوگیری نمود. در واقع با قطع مسیر پیام‌رسانی رگزایی، می‌تواند از پیدایش عروق جدید جلوگیری نماید لذا احتمالاً به‌عنوان یک عامل مناسب درمانی جدید در جلوگیری و یا کاهش رگزایی مطرح باشد.

Title: عوامل مؤثر در ارتقاي سلامت زنان سرپرست خانوار: مثلث طلایی پول، وقت و انرژی

http://knh.shmu.ac.ir/index.php/site/article/view/792

مقدمه: زنان سرپرست خانوار از گروه‌های آسیب‌پذیری هستند که به‌دلیل مواجه بودن با وضعیت اقتصادی نامناسب و مسئولیت‌های چندگانه، با مشکلات زیادی در رابطه با سلامتی خود روبرو هستند و لازم است تا هر چه بیشتر سلامت خود را ارتقاء بخشند. هدف از این مطالعه کشف و توصیف عوامل مؤثر در ارتقاء سلامت زنان سرپرست خانوار بود. مواد و روش‌ها: این مطالعه به روش کیفی و با رویکرد گراندد تئوری انجام شد. مشارکت‌کنندگان شامل 19 زن سرپرست خانوار بودند. داده‌ها از طریق مصاحبه‌های نیمه ساختارمند جمع‌آوری گردید. نمونه‌گیری به‌صورت هدفمند شروع و در طی تکامل طبقات، با نمونه‌گیری نظری ادامه یافت. برای تجزیه و تحلیل داده‌ها از روش مقایسه مداوم به شیوه اشتراوس و کوربین استفاده شد. نتایج: یافته‌های ما نشان داد که منابع باقی مانده (پول، وقت و انرژی باقی مانده) و عوامل زمینه‌ای (ویژگی‌های فردی و اقتصادی- اجتماعی) عوامل مؤثر در ارتقاء سلامت زنان سرپرست خانوار است. این زنان اغلب پس از برآوردن نیازهای مربوط‌به فرزندان/ افراد تحت سرپرستی و حل و فصل مسائل زندگی و با‌توجه به کمیت منابع باقی مانده، ممکن بود اقدام به انجام رفتارهای ارتقاء‌دهنده سلامت بزنند و این امر سلامت آنها را در معرض خطر قرار می‌داد. نتیجه‌گیری: توصیه می‌شود که دولت، سازمان‌های غیردولتی و كاركنان حرفه‌های مختلف بهداشتی با فراهم آوردن منابع موردنیاز و افزایش سطح آگاهی این زنان، تمام تلاش خود را در جهت ارتقاء سطح سلامتی این قشر آسیب‌پذیر انجام دهند.

Title: بررسی تأثير تیمار با AntimiR-182 روی افزایش حساسیت به داروی سیس پلاتین در سلول‌های Hela

http://knh.shmu.ac.ir/index.php/site/article/view/844

مقدمه: miRNA‌ها مولکول‌های RNA تك رشته‌اي غیرکدکننده به طول حدود 21 تا 23 نوکلئوتید هستند كه ترجمه نمي‌شوند و در کنترل بیان ژن نقش مهمی دارند. آنها به‌عنوان انکوژن یا تومور ساپرسور نقش مهمی در بروز برخی سرطان‌ها دارند. در گزارشات مختلف miR-182 به‌عنوان آنکومیر در سرطان گردن رحم معرفی شده است که مانع از انجام آپوپتوز در سلول می‌گردد. هدف از مطالعه حاضر بررسی تأثير AntimiR-182 در رده سلولی سرطانی Hela روی القای آپوپتوز در حضور داروی سیس پلاتین و کاهش مقاومت دارویی می‌باشد. مواد و روش‌ها: پس از کشت سلول‌های Hela با افزودن دوزهای مختلف داروی سیس پلاتین و بررسی میزان مرگ و میر سلول‌ها به‌روش MTT، میزان LD50 دارو به‌دست آمد. سپس با استفاده از حجم‌های مختلف لیپوفکتامین و miR کنترل نشاندار شده شرایط ترانسفکشن بهینه و غلظت اپتیمم AntimiR-182 محاسبه گردید. در نهایت AntimiR-182 و سیس پلاتین به‌طور مجزا و همزمان به سلول‌هایHela منتقل و میزان کاهش تکثیر سلولی و افزایش آپوپتوز ناشی از مهار به‌وسیله AntimiR-182 به تنهایی و همراه با سیس پلاتین با روش MTT و فلوسایتومتری مورد ارزیابی قرار گرفت. نتايج: نتایج حاصل بیانگر آن است که تیمار سلول‌ها با AntimiR-182 و سیس پلاتین موجب افزایش معنی‌دار مرگ سلول‌ها از 68% (تیمار با سیس پلاتین) به 81% (تیمار همزمان با AntimiR-182 و سیس پلاتین) شده است (05/0P<). این افزایش به‌صورت آپوپتوز و نکروز سلول‌ها می‌باشد. نتیجه‌گیری: مطالعه حاضر نشان می‌دهد که بیان miR-182 ممکن است در افزایش مقاومت به شیمی درمانی سلول‌های سرطانی نقش داشته باشد. استفاده از AntimiR-182 توأم با سیس پلاتین منجر به تقویت اثر سیس پلاتین و در نتیجه کاهش غلظت LD50 سیس پلاتین می‌شود، بنابراین می‌توان از آن به‌عنوان یک درمان مکمل به‌منظور افزایش حساسیت سلول‌های سرطانی به درمان و کاهش مقاومت دارویی استفاده کرد.

Title: تأتیر تجویز پروژسترون پس از تحریک تخمک‌گذاری بر میزان رگزایی و تغییرات کمی آندومتررحم موش در زمان لانه‌گزيني

http://knh.shmu.ac.ir/index.php/site/article/view/848

مقدمه: تغییر درویژگی های مورفولوژیک پارامترهای عروقی اندومتر رحم باعث تغییر در میزان لانه‌گزینی جنین می‌شود و این امر وابسته به هورمون‌های تخمدانی است. در این مطالعه به بررسی تغییرات کمی مورفولوژی اندومتر رحم پس از تزریق پروژسترون در ادامه پروتکل تحریک تخمک‌گذاری پرداخته شد. مواد و روش‌ها: در این مطالعه از موش‌های ماده بالغ استفاده شد. حیوانات به سه گروه تقسیم شدند. در گروه کنترل حاملگی کاذب القا شد و در گروه‌های تجربی، گروه تجربی 1 (گروه تحریک تخمک‌گذاری) و گروه تجربی 2 (گروه تیمار با پروژسترون پس از تحریک تخمک‌گذاری) تحریک تخمك‌گذاری انجام و سپس حاملگی کاذب القا گردید. در گروه تیمار با پروژسترون پس از تحریک تخمک‌گذاری، متعاقب القا حاملگی کاذب روزانه تزریق mg/mouse 1 پروژسترون صورت گرفت. سپس در زمان لانه‌گزيني از هر سه گروه جهت تعیین سطح هورمون‌های تخمدانی خونگیری از قلب انجام شد سپس نمونه‌برداری از بافت رحم جهت مطالعات بافتی صورت گرفت. پس از جمع‌آوری داده‌ها بررسی آماری انجام شد. نتایج: یافته‌ها در گروه‌های تجربی افزایش معني‌دار آماری در سطح هورمون پروژسترن، تعداد مقاطع عروق، مساحت داخلی عروق و قطر داخلی لومن غدد نسبت‌به گروه کنترل نشان داد (05/0P≤). همچنین مقایسه بین گروه تجربی 1 و 2 نشان داد که در گروه تجربی 2، تزریق پروژسترون پس از تحریک تحمک‌گذاری باعث افزایش معني‌دار آماری در سطح هورمون پروژسترون و تعداد مقاطع عروق شده است (05/0P≤). نتیجه‌گیری:تزریق پروژسترون پس از تحریک تخمك‌گذاری شرایط مورفولوژی آندومتر رحم را نسبت‌‌به گروه تحریک تخمك‌گذاری بهبود می‌بخشد و این امر احتمالاً باعث افزایش موفقیت لانه‌گزيني خواهد شود.

Title: مقایسه سه شیوه مختلف تمرین موازی بر سطوح پلاسمایی کمرین، مقاومت به انسولین و عملکرد جسمانی زنان سالمند

http://knh.shmu.ac.ir/index.php/site/article/view/867

مقدمه: تمرین ترکیبی به علت اختصاصی بودن اثرات تمرین، برای عملکرد بدنی مطلوب و سلامتی در افراد سالمند توصیه شده است. هدف پژوهش حاضر مقایسه سه شیوه مختلف تمرین موازی بر سطح پلاسمایی کمرین، مقاومت به انسولین و عملکرد جسمانی زنان سالمند بود. مواد و روش‌ها: این مطالعه از نوع نیمه تجربی بود که در آن 40 زن سالمند به‌صورت هدفمند انتخاب شدند و به‌طور تصادفی به 4 گروه، تمرین مقاومتی- استقامتی (10 نفر)، تمرین استقامتی- مقاومتی (9 نفر)، تمرین اینتروال (12 نفر) و کنترل (9 نفر) تقسیم شدند. آزمودنی‌های گروه تجربی 8 هفته و هر هفته سه جلسه تمرین داشتند. از آزمون t و واریانس یک طرفه جهت مقایسه میانگین متغیرها استفاده گردید. نتایج: نتایج نشان داد که در گروه‌های تجربی پس از هشت هفته تمرین مقادیر کمرین (300/0=P)، انسولین (72/0=P)، گلوکز ناشتا (09/0=P)، مقاومت به انسولین (69/0=P)، درصد چربی (08/0=P) و WHR (55/0=P) تغییر معنی‌دار پیدا نکرد. اما کاهش معنی‌دار در وزن (017/0=P) و BMI (023/0=P) مشاهده شد. نتیجه‌گیری: بر اساس یافته‌های این مطالعه به‌نظر می‌رسد آرایش و ترتیب تمرین ترکیبی سازگاری‌های متابولیکی و کمرین را تحت تأثیر قرار نمی‌دهد اما هر سه نوع تمرین می‌توانند منجر به تغییرات متفاوتی نسبت‌به قبل از دوره تمرین شوند.

Title: بررسی وضعیت کیفی آب‌های زیرزمینی با استفاده از شاخص GWQI و پهنه‌بندی آن با سامانه اطلاعات جغرافیایی (GIS)

http://knh.shmu.ac.ir/index.php/site/article/view/878

مقدمه: باتوجه به آنکه منابع آب زیرزمینی عمده‌ترین منابع تأمین آب شهرستان گرمی به‌شمار می‌رود لذا اطمینان از مطلوبیت آن به‌منظور حفظ سلامت مصرف‌کنندگان، اهمیت فراوانی دارد لذا هدف این مطالعه، بررسی وضعیت کیفی آب‌های زیرزمینی منطقه گرمی با استفاده از شاخص GWQI (شاخص کیفی آب زیرزمینی) و پهنه‌بندی آن با سامانه اطلاعات جغرافیایی (GIS) می‌باشد. مواد و روش‌ها: این مطالعه از نوع مطالعات توصیفی تحلیلی می‌باشد که به مدت 14 ماه بر روی منابع آب زیرزمینی شهرستان گرمی انجام شد. برای این منظور، عملیات نمونه‌برداری از آب تمامی این چاه‌ها به مدت یک سال به‌صورت ماهی یک بار و در فاصله زمانی آذر ماه 1392 تا آذر ماه 1393 از طريق شير برداشـت خروجـي از چـاه صورت گرفت. سپس نمونه‌ها به سرعت به آزمایشگاه شرکت آب و فاضلاب استان اردبیل منتقل و مقدار شاخص‌های فیزیکو شیمیایی موردنیاز برای تعیین شاخص GWQI شامل pH، جامدات محلول کل، کلرور، کلسیم، منیزیم، سولفات، بیکربنات و سدیم با استفاده از روش‌های ذکر شده در کتاب استاندارد متد 2008 تعیین و براساس آن مقدار این شاخص محاسبه شد. همچنین با استفاده از سامانه اطلاعات جغرافیایی، نقشه پهنه‌بندی کیفی آب‌های زیرزمینی شهرستان گرمی براساس شاخص GWQI تهیه شد. نتايج: نتایج مطالعه نشان داد که مقدار شاخص GWQI در آب‌های زیرزمینی شهرستان گرمی بین 37/41 تا 86/44 می‌باشد و آب منطقه در محدوده خوب قرار دارد. همچنین مقدار این شاخص برای چاه ارانچی، کمترین (37/41) (بالاترین کیفیت) و برای چاه گوده‌ کهریز، بیشترین (86/44) (پايین‌ترین کیفیت) می‌باشد. در این مطالعه، مقدار تمامی شاخص‌هاي کیفی آب غیر از جامدات محلول کل در حد استاندارد بود. نتیجه‌گیری: براساس نتایج این مطالعه، کیفیت آب‌های زیرزمینی شهرستان گرمی در حد خوب قرار دارد ولی مقدار کلسیم و منیزیم آن هر چند در محدوده استاندارد واقع است اما در سطوح پايینی قرار دارد و باتوجه به نیاز بدن به این ریزمغذی‌ها، باید برنامه‌ریزی لازم در خصوص تأمین این عناصر از طریق سایر منابع یا افزودن آنها به آب در تصفیه‌خانه صورت گیرد.

Title: بررسي تأثير هشت هفته تمرين منظم هوازي و مصرف عصاره گزنه برسطح گلوکز و شاخص مقاومت به انسولين زنان ديابتي نوع دو

http://knh.shmu.ac.ir/index.php/site/article/view/886

مقدمه: هدف تحقيق حاضر بررسي تأثير هشت هفته تمرين منظم هوازي و مصرف عصاره گزنه برسطح گلوکز و شاخص مقاومت به انسولين در زنان ديابتي نوع2 است. مواد و روش‌ها: اين مطالعه بر روي 46 زن از بين کليه زنان ديابتي نوع 2 که در انجمن ديابت شهرستان شاهرود پرونده داشتند، انجام شد. پس از کسب رضايت‌نامه و اقدامات اوليه لازم، آرمودني‌ها به‌صورت کاملاً تصادفي در چهار گروه: 1) گروه تمرين هوازي و مصرف مکمل گزنه (13 نفر)، 2) گروه تمرين و مصرف دارونما (13 نفر)، 3) گروه مصرف مکمل گزنه (13 نفر)، 4) 7 نفر گروه کنترل تقسيم و مورد مطالعه قرار گرفتند. تمرينات هوازي شامل فعاليت به مدت 8 هفته، هفته‌اي سه جلسه‌ي 30 تا 55 دقيقه‌اي با شدت 45 تا 65 درصد ضربان قلب ذخيره بود. مصرف مکمل روزي 6 سي سي عصاره گزنه شرکت اسانس گياه گرگان به‌صورت محلول در آب استفاده شد و دارونما روزي 6 سي سي محلول دارونما (آب و رنگ مشابه عصاره) بود. نمونه‌هاي خون قبل و بعد ار دوره تمريني و مکمل‌گيري به‌منظور اندازه‌گيري سطح گلوکز و شاخص مقاومت به انسولين گرفته شد. براي تجزيه و تحليل يافته‌ها از نرم‌افزارSPSS  و روش تحليل واريانس استفاده گرديد. نتايج: هشت هفته تمرين هوازي و مصرف گياه گزنه برسطح گلوکز خون و شاخص مقاومت به انسولين زنان سالمند ديابتي نوع 2 تأثير معناداري نداشتند (05/0<P). نتيجه‌گيري: تمرين هوازي و مصرف گياه گزنه با اين شدت و مقدار بر سطح گلوکز خون و شاخص مقاومت به انسولين زنان سالمند ديابتي نوع 2 تأثير معناداري نداشت و اين بيماران براي بهره‌برداري مؤثر از تمرين هوازي و مصرف گياه گزنه، تمرين‌ها را با شدت و مدت بيشتر انجام دهند و گزنه را با دوز بالاتر مصرف نمايند.

Title: چهار مرحله تمايز سلول‌هاي بنيادي مزانشيمي حاصل از بندناف به هپاتوسيت‌ها در داربست هيدروژلي

http://knh.shmu.ac.ir/index.php/site/article/view/908

مقدمه: باتوجه به افزايش تقاضا براي پيوند كبد و كمبود اهدا كنندگان عضو، توليد بافت كبد از طريق روش‌هاي مهندسي بافت كبد و كشت سه بعدي مورد بررسي قرار گرفته است. مطالعه حاضر با هدف جداسازی، استخراج و تمایز سلول‌های بنیادی مزانشیمی بند ناف به هپاتوسیت‌ها و ارزیابی مارکرهای سلولي و بررسی رفتار و عملكرد آنها طراحی گردید مواد و روش‌ها: سلول‌هاي بنيادي مزانشيمي از بندناف استخراج و جداسازي شده بررسی مارکرها و فنوتيپ سلول‌های بنیادی از طریق فلوسیتومتری انجام شد. سپس در طی یک دوره 14 روزه، سلول‌ها تحت چهار مرحله تمایز با عامل رشد فیبروبلاستی 4(FGF4)، عامل رشد هپاتوسیتی (HGF)، دگزامتازون (DEX)، انسولین-ترانسفرین-سلنیوم (ITS)، گلوکاگون (GLU) انکوستاتین(OSM)  و تریکواستاتین (TSA) قرار گرفتند. به‌وسيله اليزا توليد اوره  توسط سلول‌ها اندازه‌گيري شد. جهت بررسي بافت‌شناسی تمايز، مقاطع كشت سه بعدي تهيه شده، به‌وسيله روش هماتوکسیلین/ ائوزین رنگ‌آمیزی شد. نتايج: سلول‌هاي حاصل از ژله وارتون ماركرهاي سطحي مزانشيمي مانند CD73 را نشان داند اما اين سلول‌ها فاقد ماركرهاي سطحي سلول‌هاي خونساز مانند CD31 بودند. پس تمايز چند مرحله‌اي سه بعدي، مورفولوژي سلول‌ها گرد شده، در داربست آلژيناتي تجمعات سلولي به‌دليل نزديك شدن سلول‌ها ديده شد. مقادير اوره در هر دو كشت سه بعدي و دو بعدي به‌طور معني‌داري در الگوي وابسته به زمان افزايش يافت. مقادير اوره در كشت سه بعدي نسبت‌به كشت دو بعدي بيشتر بود (001/0P=). با بررسي بافت‌شناسی مقاطع دركشت سه بعدي بافتي پرسلول با دستجات و صفحات سلولي با هسته يوكروماتين ديده شد. نتيجه‌گيري: نتايج اين مطالعه نشان داد كه تمايز 4 مرحله‌اي سلول‌هاي بنيادي با استفاده از داربست هيدروژلي باعث ظهور ويژگي‌هاي عملكردي و مورفولوژيكي سلول‌هاي هپاتوسيتي مانند ترشح اوره و ايجاد صفحات سلولي شبه كبدي مي‌گردد.

Title: ارزیابی میزان آنتي‌بادي اختصاصي بر عليه آنتي‌ژن نوتركيب ESAT-6 مايكوباكتريوم توبركلوزيس در تشخيص توبركلوزيس گاوي

http://knh.shmu.ac.ir/index.php/site/article/view/939

مقدمه: توبركلوزيس گاوي نوعي بيماري مشترك بين انسان و حيوان است. عامل اين بيماري مايكوباكتريوم بوويس مي‌باشد. روش‌هاي تشخيص رايج اين بيماري شامل سنجش سطح اينترفرون گاما و آزمون جلدي مي‌باشد كه هر كدام از آنها معايب خاص خود را دارند اما روش‌هاي سرولوژيك مبتني بر الايزا توانسته‌اند نويدبخش تشخيص سريع و مناسب بيماري سل گاوي باشند. در اين مطالعه بررسي اهميت آنتي‌ژن نوتركيب ESAT-6 به‌عنوان هدفي براي رديابي آنتي‌بادي‌هاي اختصاص موجود عليه اين پروتئين به‌منظور تشخيص دقيق بيماري سل گاوي به‌روش الايزا مي‌باشد. مواد و روش‌ها: در اين مطالعه نمونه‌هاي سرمي از 21 گاو آلوده‌اي كه بيماري در آنها به‌وسيله آزمون جلدي و معاينات باليني تأييد شده بود و 20 دام سالم (به‌عنوان كنترل) جمع‌آوري شد و ميزان آنتي‌بادي اختصاصي بر عليه آنتي‌ژن نوتركيب ESAT-6 به‌وسيله روش الايزاي طراحي شده مورد ارزيابي قرار گرفت. نتايج:‌ نتايج به‌دست آمده همخواني تقريباً‌ قابل قبولي با نتايج آزمون جلدي دارد. ميزان حساسيت و ويژگي اين روش در رقت سرمي يك صدم به‌ترتيب 23/95 و 87/87 به‌دست آمد. نتيجه‌گيري:‌ نتايج اين مطالعه به‌خوبي بيانگر اين نكته است كه روش الايزاي مبتني بر ارزيابي ميزان آنتي‌بادي بر عليه ESAT-6 مي‌تواند به‌عنوان يك روش تشخيص مناسب براي بيماري سل گاوي در نظر گرفته شود كه با استفاده از اين روش مي‌توان نمونه‌هاي آلوده را به‌طور دقیق و سریع مورد ارزيابي قرار داد.
